# ROUTINE USE OF V-LOCK^®^ SUTURE FOR BARIATRIC ANASTOMOSIS IS SAFE: COMPARATIVE RESULTS FROM CONSECUTIVE CASE SERIES

**DOI:** 10.1590/0102-672020190001e1452

**Published:** 2019-10-21

**Authors:** Claudia BURES, Philippa SEIKA, Christian DENECKE, Johann PRATSCHKE, Ricardo ZORRON

**Affiliations:** 1 Center for Bariatric and Metabolic Surgery, Center of Innovative Surgery (ZIC), Department of Surgery, Campus Virchow Klinikum and Campus Mitte, Charité-Universitätsmedizin Berlin, Berlin, Germany;; ² Center for Bariatric and Metabolic Surgery, Klinikum Ernst von Bergmann Potsdam, Potsdam, Germany

**Keywords:** Laparoscopy, Bariatric surgery, Sutures, Anastomosis, surgical, Suture techniques, Laparoscopia, Cirurgia bariátrica, Sutura, Anastomose cirúrgica, Técnicas de sutura

## Abstract

**Background:**

In high-income countries, morbid obesity is a growing health problem that has already reached epidemic proportions. When performing a laparoscopic gastric bypass several operative methods exist.

**Aim:**

To describe the institutional experience using a knotless unidirectional barbed suture (V-Loc 180/Covidien, Mansfield, MA) to create a hand-sewn gastrojejunostomy (GJ) and jejunojejunostomy (JJ) during bariatric surgery.

**Methods:**

Evaluation of a case series of 87 morbidly obese patients who underwent laparoscopic gastric bypass with a hand-sewn gastrojejunostomy (GJA) and jejunojejunostomy (JJA) between 01/2015 and 06/2017. The patients were divided into two groups: in group I, GJA und JJA sutures were performed using the knotless unidirectional barbed suture; in group II, GJA and JJA were sutured with resorbable multifilament thread (Vicryl^®^ 3/0 Ethicon, Livingstone, UK). The recorded data on gender, age, BMI, ASA score, operative time, postoperative morbidity, length of hospital stay, and reoperation, were analyzed and compared.

**Results:**

All procedures were completed laparoscopically with no mortality. The mean operative time was 123.23 (±30.631) in group I and 127.57 (±42.772) in group II (p<0.05). The postoperative complications did not differ significantly between the two groups. Early complications were observed for two patients (0.9%) in the barbed suture group and for one patient (0.42%) in the multifilament suture group (p<0.05). In group I two patients (0.9%) required reoperation: on the basis of jejunojejunal stenosis in one patient, and local abscess near the gastrojejunostomy, without a leakage, in the other. In group II one patient (0.42%) required reoperation due to stenosis of the GJA. The duration of hospital admission was similar for both groups: 3.36 (±0.743) days in group I vs. 3.38 (±1.058) days in group II (p<0.05).

**Conclusion:**

The novel anastomotic technique is a safe and effective method and can be applied to gastrojejunal anastomosis and jejunojejunal anastomosis in laparoscopic gastric bypass.

## RESUMO

**Racional:**

*:* Em países de alta renda, a obesidade mórbida é um problema crescente de saúde que já atingiu proporções epidêmicas. Ao realizar um bypass gástrico laparoscópico, existem vários métodos operatórios.

**Objetivo:**

Descrever a experiência institucional utilizando uma sutura farpada unidirecional sem nós (V-Loc 180/Covidien, Mansfield, MA) para criar gastrojejunostomia (JJ) e jejunojejunostomia (JJ) costuradas à mão durante a cirurgia bariátrica.

**Métodos:**

Avaliação de uma série de casos com 87 pacientes obesos mórbidos submetidos ao bypass gástrico por videolaparoscopia com gastrojejunostomia (JJA) e jejunojejunostomia (JJA) suturados à mão entre 01/2015 e 06/2017. Os pacientes foram divididos em dois grupos; no grupo I, GJA e JJA as suturas foram realizadas com a sutura farpada unidirecional sem nós e, no grupo II, com sutura multifilamentar reabsorvível (Vicryl^®^ 3/0 Ethicon, Livingstone, UK). Foram analisados ​​e comparados os dados registrados sobre gênero, idade, IMC, escore ASA, tempo operatório, morbidade pós-operatória, tempo de internação hospitalar e reoperação.

**Resultados:**

Todos os procedimentos foram concluídos por laparoscopia sem mortalidade. O tempo cirúrgico médio foi 123,23 (±30,631) no grupo I e 127,57 (±42,772) no grupo II (p<0,05). As complicações pós-operatórias não diferiram significativamente entre os dois grupos. Complicações precoces foram observadas em dois pacientes (0,9%) no grupo de sutura farpada e um (0,42%) no de sutura multifilamentar (p<0,05). No grupo I, dois pacientes (0,9%) necessitaram de reoperação; um devido à estenose jejunojejunal e abscesso local próximo à gastrojejunostomia, sem vazamento, no outro. No grupo II, um paciente (0,42%) necessitou de reoperação por estenose da GJA. O tempo de internação hospitalar foi semelhante nos dois grupos: 3,36 (±0,743) dias no grupo I vs. 3,38 (±1,058) dias no grupo II (p<0,05).

**Conclusão:**

A nova técnica de anastomose é método seguro e eficaz e pode ser aplicado nas anastomoses gastrojejunal e jejunojejunal no bypass gástrico laparoscópico.

## INTRODUCTION

In high-income countries, morbid obesity is a growing health problem that has already reached epidemic proportions^1^ ^-^ ^3^. Bariatric surgery is considered the only effective long-term treatment for morbid obesity^4^ ^-^ ^6^. When performing a laparoscopic gastric bypass in particular during the construction of gastrojejunostomy (GJA) and jejunojejunostomy (JJA), several operative methods exist. Although different types of anastomotic techniques are available (hand-sewn, linear-stapled and circular-stapled anastomosis), the choice of technique when performing these anastomoses is based mostly on personal preference. The intestinal anastomosis is one of the most complex and time-consuming procedures in laparoscopy^12^ and laparoscopic intracorporeal suturing and knot tying for anastomosis are considered difficult laparoscopic skills to master. Furthermore, a knot can be a source of anastomotic failure^9^ ^-^ ^13^. 

The use of barbed sutures for laparoscopic surgery have the potential advantage of performing the anastomosis without the need of knotting the sutures, possibly leading to a shorter learning curve. V-Loc 180^®^ is a barbed absorbable thread armed with a surgical needle at one end and a loop at the other end, which is used to secure the suture. The barb and loop-ends make it possible to approximate the tissues without the need for surgical knots. However, the routine use of barbed sutures without knotting has not yet proven safe for gastrointestinal anastomosis and therefore, has yet to be routinely applied as the standard technique. 

In this study, we monitored a series of 87 consecutive patients who were operated in our medical center for treatment of morbid obesity, and compared two anastomotic techniques: unidirectional absorbable barbed suture (3-0 V-Loc 180^®^, Medtronic, Mansfield, MA, USA) and absorbable suture (3-0 Vicryl^®^) with laparoscopic knotting. 

The aim of this study was to describe the results of the institutional experience in a comparative evaluation of both suturing methods in hand-sewn gastrojejunostomy (GJ) and jejunojejunostomy (JJ) during bariatric surgery

## METHODS

This study is a prospective documentation of Roux-en-Y gastric bypasses (RYGB) from January 2015 to June 2017 which were operated in our center. From December 2016 onwards, all patients received a GJ and JJ anastomosis performed with V-Loc 180^®^. All operations were carried out in a standardized setting by three experienced bariatric surgeons (more than 500 laparoscopic surgical procedures).

A series of 87 consecutive patients operated in our medical center for treatment of morbid obesity, were prospectively documented for the use of laparoscopic hand-sewn closure of the gastrojejunostomy (GJA) and a jejunojenuostomy (JJA). In group I, we used a unidirectional absorbable barbed suture (V-Loc 180^®^, Medtronic, Mansfield, MA, USA) to perform anastomosis; in group II, we performed anastomosis with an absorbable suture (Vicryl 3/0^®^, Ethicon, Livingstone, UK ) making an intracorporeal knot. V-Loc 180^®^ is a barbed absorbable thread armed with a surgical needle at one end and a loop at the other end, which is used to secure the suture. This technology enables the approximation of tissues, making surgical knots redundant.

Outcomes were assessed through multivariate analysis, adjusting for gender, age, BMI, co-morbidity (ASA/American Society of Anaesthesiology score), operative time, postoperative morbidity, length of hospital admission, and number of reoperations (Table 1).

**TABLE 1:** Preoperative characteristics (group I barbed suture, group II multifilament suture)

Data	Group I	Group II
Number of patients (n=87)	45 (51.7%)	42 (48.3%)
Gender (m=21/w=66) male	11 (24.4%)	10 (23.8%)
female	34 (75.6%)	32 (76.2%)
Age (years)	43.74 ±10.918	45.45 ±12.609
BMI/ Body mass index (kg/m²)	47.71 ± 6.634	46.42 ±7.284
ASA Score 1	3 (6.7%)	2 (4.8%)
2	26 (57.8%)	22 (52.4%)
3	16 (35.6%)	18 (42.9%)
Operation primary	44 (97.8%)	31 (73.8%)
Re-do	1 (2.2%)	11 (26.2%)

p value not significant

### Operative technique

**FIGURE 1:**
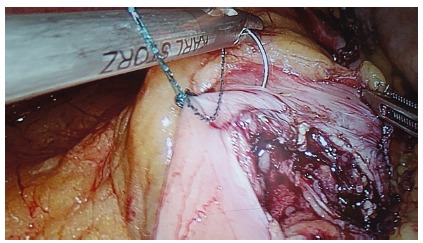
Gastrojejunal anastomosis using barbed suture: approaching a pre formed loop to fix the suture proximally, avoiding the need of knotting; the suture starts 5 mm proximal to the defect to allow safe closure.

**FIGURE 2:**
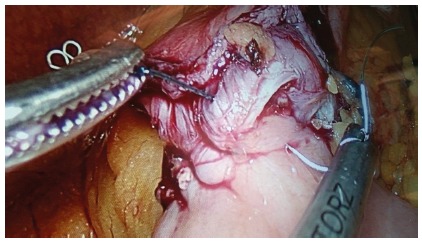
Progress of the suture with seromuscular running suture: the anastomosis is calibrated with a 36 Fr bougie; each catch in the tissue has to be firmly drawn to permit a tight anastomosis.

**FIGURE 3:**
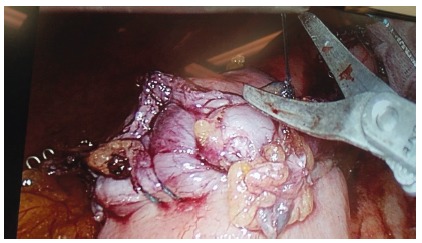
Suture ending: at 5 mm after the anastomosis end in already stapled tissue to ensure safe and full tight suture; no clip is needed to ensure the distal end of the suture.

**FIGURE 4:**
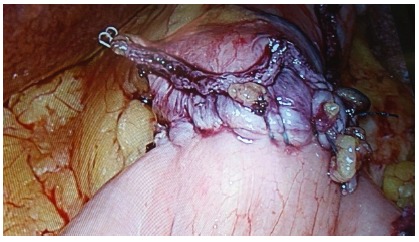
Leak test of the anastomosis is routinely performed with instillation of methylene blue solution through the transoral bougie.

Laparoscopic gastric bypass is a standardized procedure. Between four to five ports were used to perform the procedure, three 12 mm ports and one to two 5 mm ports. We began by inflating the abdomen to 15 mmHg intra-abdominal pressure. The gastric sections were made by using a 60 mm linear stapler, the length of the biliopancreatic limb measured 50 cm from the ligament of Treitz, the Roux limb was measured at 150 cm. The jejunojejunostomy was created in side-to-side fashion using a 60 mm EndoGIA linear stapler with a staple height of 2.5 mm. The enterotomy was closed with a barbed suture (V-Loc 180^®^) in group I, while in group II a resorbable polyfilament suture (Vicryl 3/0^®^) was used (Figures 1 to 4). A 50 ml gastric pouch was created using a 60 mm linear EndoGIA stapler with a staple height of 3.5 mm. An antecolic antegastric approach to anastomosis of the Roux limb and gastric pouch was used; the gastric pouch was established around a 36 Fr gastric tube. The gastrojejunostomy was performed antegastrically using a 60 mm linear EndoGIA stapler with a staple height of 3.5 mm. One layer running suture with barbed suture (V-Loc 180^®^) was created to close the gastrojejunal anastomosis in group I while a resorbable polyfilament suture (Vicryl 3.0^®^) was used in group II. In both groups the mesenteric windows were closed with a continuous non-resorbable suture (Ethibond 0^®^, Ethicon, Livingstone, UK). An intraoperative leak test was performed for all patients by insufflating the pouch and anastomosis with 100 ml methylene blue under pressure. No drain was used.

### Statistical analysis

Data were collected prospectively using a computerized database. Quantitative data were given as median (range). Difference between the groups was accessed by Mann-Whitney and X^2^ tests or Fischer exact test when appropriate. Statistical significance was defined as p<0.05.

## RESULTS

Between 01/2015 and 06/2017 87 morbidly obese patients underwent laparoscopic gastric-bypasses using a hand-sewn technique to perform the gastro-jejunal anastomosis (GJA) and jejunojenunal anastomosis (JJA).

The study population comprised 66 (75.9%) women and 21 (24.1%) men with an average age of 44.630 (±11.78) years and an average BMI of 47.089 (±6.95). There were no significant differences between these two groups of patients with respect to age, gender, BMI, and ASA (Table 1). 

Group I included 34 (75.6%) female and 11 (24.4%) male; group II 32 (76.2%) female and 10 (23.8%) male. The average BMI was 47.711 (±6.63) for group I and 46.421 (±7.28) for group II, with no significant differences. Similarly, there were no significant differences regarding the patients’ age, 43.74 (±10.918), in group I compared to 45.45 (±12.609) in group II. 

In group I, 44 patients (97.8%) underwent a primary Roux-en-Y gastric bypass, on one (2.2%) a redo-operation was carried out after sleeve-gastrectomy. Group II comprised 42 patients: 31 (73.8%) underwent a primary Roux-en-Y gastric bypass and 11 (26.2%) redo-operations after sleeve-gastrectomy. In group I, a simultaneous hiatal hernia repair and cholecystectomy was performed on one patient; cholecystectomy was carried out on three patients; and one underwent adrenalectomy and cholecystectomy. In group II, simultaneous cholecystectomy was performed on one patient.

Mean operative time was 123.23 (±30.631) min in group I (V-loc procedures) compared to 127.57 (±42.772) min in group II (Vicryl-procedures). The average hospitalization duration was 3.36 (±0.743) days in group I and 3.38± (1.058) days in group II. No conversion to open procedures occurred in either of these groups (Table 2). 

**TABLE 2:** Perioperative characteristics (group I barbed suture, group II multifilament suture)

Data	Group I	Group II
Operation time (minutes)	123.23 (±30.631)	127.57 (±42.772)
Conversion	0	0
Complication	2 (0.9%)	1 (0.42%)
	* stenosis of JJA	* stenosis from GJA
	* local abscess near GJA	
Re-operation	2 (0.9%)	1 (0.42%)
Length of hospital stay (days)	3.36 (±0.743)	3.38± (1.058)
Mortality	0	0

Postoperative complications did not differ significantly between these two groups. All patients were monitored postoperatively for 30 days. Two patients in group I and one in group II had operative revisions. In group I, one stenosis of jejunojejunal anastomosis was recorded and one patient developed a local abscess without anastomosic leakage that was drained. In group II, one stenosis from gastrojejunostomy was documented. There were no anastomotic leakages, fistulae, or bleedings in both groups. The 30-day mortality rate was zero in both groups. 

## DISCUSSION

The intestinal application of barbed suture has not been accepted as a routinely standard method for gastrointestinal anastomosis yet. Laparoscopic intracorporeal suturing and knot tying are considered to be the most difficult laparoscopic skills to master. Furthermore, a knot can be a source of failure^7^ ^-^ ^11^. 

Laparoscopic digestive anastomosis is a technically demanding procedure that requires advanced skills in laparoscopic surgery. Moreover, its complications are responsible for a large proportion of the ensuing mortality and morbidity (leak, fistula, intraabdominal abscess, stenosis). The knotless barbed suture has been proposed to make laparoscopic suturing easier.

In 1956, Dr. J. H. Alcamo was granted the first patent for a unidirectional barbed suture^13^. The procedure was used for the repair of flexor tendons in the hand and was the first published study of this kind^14^. In 2009, Demyttenaere et al.^7^ published a randomized study in 12 pigs comparing enterotomy closure with barbed vs. non-barbed suture. There are a few experimental studies assessing the benefits of a barbed suture; however, they cannot be related directly to general surgery. In 2013 and 2014, five studies presented the results of gastric bypasses using a barbed suture for gastrojejunostomy compared to the conventional knot-tying anastomotic technique. Milone et al.^20^ reported the first prospective randomized controlled study evaluating the efficacy of V-Loc suture for gastrojejunal anastomosis on 60 patients undergoing mini gastric-bypass when a barbed suture was used on 30 patients, while on the other 30 Polysorb 3/0 was used. The results show that the knotless unidirectional barbed suture does not only show a reduction in the time required to close the anastomosis but, also, reduced costs compared to the conventional knot-tying anastomosis. There were no significant differences in total operative time and regarding complications.

De Blasi et al.^9^ compared 100 candidates who underwent Roux-en-Y gastric-bypasses; in 50 gastrojejunostomy was sutured with resorbable interrupted suture, while on the other 50 a continuous barbed suture was used. This study showed that the use of barbed sutures was as safe as conventional sutures and allowed easier and faster sutures in the creation of gastrojejunostomy. The anastomotic time was shorter, but not significantly reducing the total operative time.

Tyner et al.^18^ showed in a retrospective review that there are no differences in the 30-days outcomes between knotless unidirectional barbed sutures and absorbable sutures with knots. In the study, 38 gastric-bypass operations with a traditional technique two-layer anastomosis were compared to 46 operations with the innovative technique using a knotless unidirectional barbed monofilament absorbable suture. Unidirectional barbed sutures can be safely used for GJ anastomosis. Constantino et al.^19^ showed in a prospective study with 239 Roux-en-Y gastric-bypasses that barbed suture/V-Loc 180^®^ is a safe procedure and reduces the operation time. This study recorded operative time, the time used for anastomosis construction, the conversion rate, and all kinds of complications**.** Palmisano et al.^21^ published a study regarding 96 hand-sewn gastrojejunostomy and jejunojejunal anastomosis using V-Loc 90^®^ barbed running suture in two layers with placing an absorbable clip at the distal end. Two leaks occurred in the jejunojejunal anastomosis and none in the gastrojejunal anastomosis. The data demonstrates the safety and effectiveness of the barbed suture procedure.

This brief review of previous clinical studies also shows that the gastrojejunal anastomosis (GJA) and jejunojejunal anastomosis (JJA) using linear stapler or completely hand-sewn are safe and reproducible when performed by an experienced surgeon^15^ ^,^ ^16^ ^,^ ^17^. There are no significant differences regarding morbidity and number of reoperations.

In concordance with the current literature, the present study shows that the use of a barbed suture is as safe as regular sutures for the closure of gastrojejunostomy and jejunojejunostomy during laparoscopic Roux-en-Y gastric-bypasses in terms of postoperative complications during the first 30 days. The innovative anastomosic technique does not reduce significantly the total operative time. Few complications were noted in both groups. Although this study compares two consecutive groups without a randomization, barbed suture appears to be easier to perform and to teach. One limitation of a barbed suture is the impossibility to remove the entire suture once inserted; the usual solution to extract an existing barbed suture is by cutting through the suture in different places and removing in pieces. While the time for the anastomosis is shorter, the total operative time is not reduced significantly.

## CONCLUSION

Due to its safety profile and ease of management, this kind of suture can be included in the standard surgical bariatric technique and can help attending surgeons to train residents in the difficult task of gastrointestinal anastomosis. 
